# Lifetime distribution of clusters in binary mixtures involving hydrogen bonding liquids

**DOI:** 10.1038/s41598-022-12779-0

**Published:** 2022-06-01

**Authors:** Ivo Jukić, Martina Požar, Bernarda Lovrinčević, Aurélien Perera

**Affiliations:** 1 Laboratoire de Physique Théorique de la Matière Condensée (UMR CNRS 7600), Sorbonne Université, 4 Place Jussieu, 75252 Paris Cedex 05, France; 2 Department of Physics, Faculty of Science, University of Split, Ruđera Boškovića 33, 21000 Split, Croatia; 3 Doctoral School of Biophysics, Faculty of Science, University of Split, Split, Croatia

**Keywords:** Chemistry, Physical chemistry, Chemical physics

## Abstract

Hydrogen bonded liquids are associated liquids and tend to exhibit local inhomogeneity in the form of clusters and segregated sub-nano domains. It is an open question as to whether Hbonded clusters in pure water have common features with the water segregated pockets observed in various aqueous binary mixtures, such as water–alcohol mixtures, for example. In the present study, we demonstrate through classical molecular dynamics studies of the lifetime distributions of the hydrogen bonds in different types of binary mixtures, that these lifetimes exhibit the same universal features in the case of the pure liquids, independently of the species concentrations. The same types of three distinct lifetimes are observed, all of them in the sub picosecond regime. The primary lifetime concerns that of Hbonded dimers, and strongly depends on Hbonding criteria such as the bonding distance. The two others are independent of bonding criteria and appear as universal accross many liquids and mixtures. The secondary lifetime ($\tau _1 \approx 20$ fs) concerns Hbonded cluster lifetimes, while the tertiary lifetime ($\tau _2 \approx 50$ fs) concerns the topology of these clusters, such as chains or globules, for example. This surprizing separation in three distinct lifetimes suggests the existence of associated three distinct kinetic mechanisms in the very short sub-picosecond time scales, with, in addition, an appealing connection to the concepts of local energy and entropy.

## Introduction

Hydrogen bonding is an important physical process, occurring in many different contexts, ranging from chemical systems^1^ to pharmaceutical^2–5^ and biological systems^6^. It links proton donor and proton acceptor molecules through a quantum mechanical process^7^, creating a labile molecular entity, which is both fragile and robust to the surrounding thermal disruptive agitation. It is robust enough to allow newly formed labile molecules to play an important role in the system, and fragile enough so that this role is only temporary. In that, it permits the appearance and destruction of transient molecular entities, but also specific architectures such as DNA, for example. Hence, the lifetime of such labile transient structure is an important physical parameter. In order to study it using statistical physics, one can define through the lifetime $\tau _{ij}$ of the Hbond between 2 molecules *i* and *j*, a microscopic random variable by $h_{ij} (t)= \delta ( t - \tau _{ij} )$ . This statistics can be studied conveniently, for example by computer simulations. Quantities such as the average, or the auto-correlation of this variable, can be computed. Interestingly, only the correlations have been the focus of previous studies^8–11^. In a recent work^12^, based on a classical description of Hbonding through Coulomb charge association, we have examined the lifetime *L*(*t*) of the Hbond, which is related to the average of $h_{ij}(t)$, for several Hbonding molecules, such as water, alcohols and amines, defined as follows:

1 $$\begin{aligned} L(t)=\frac{1}{\Lambda }\sum _{ij\in {\mathcal {C}}}h_{ij}(t) \end{aligned}$$

where $\Lambda$ is defined such that the normalisation condition $\int _0 ^{T_0} dt L(t)=1$ holds, when $T_0$ is the time window of measurement. Additional details are provided in the SI document. ${\mathcal {C}}$ is the ensemble of bound atoms which obey assigned geometrical bonding criteria such as the bonding distance $r_c$ and the bonding angle $\theta _{ij}=\widehat{HO_{i}O_{j}}$ between the two hydrogen bonded oxygen atoms $O_i$ and $O_j$. This latter constraint is defined as usual as $\theta _{ij} \le 30$.

We have uncovered an unexpected interesting universality in the lifetime distribution across these very different molecules, which appears at very short times in the sub-pico second regime. We have provided arguments which support the fact that cluster formation is the reason for this universality, all clusters being built by assembling pairs of bonded molecules into larger entities. Additionally, we have shown that the long time kinetics were system specific, in contrast to the very short time behaviour. This is a non-intuitive behaviour, since it is generally believed that it is the long time kinetics which should have universal features since one reaches the macroscopic limit^8^. The fact that the study of Hbond links to the existence of cluster formation is an interesting reductionism, in the sense that it brings an important problem in physical chemistry into the realm of clustering and aggregate formation, which is well known in associating liquids^13–16^ and mixtures^17–21^.

In the present work, this study is extended to the cases of mixtures, both for the case of two associated liquids, and those with a non-associating solute partner. Typically, we will consider aqueous alcohol and aqueous–amine mixture for the first case, aqueous DMSO, alcohol acetone and alcohol alkane for the second type. All such mixtures are typical examples of molecular emulsions^22,23^, and are know to present sub-nano scale inhomogeneity. While one would expect marked concentration dependence between neat liquids and various mixtures, precisely because of the local segregation of the species, our results suggest a surprizing similarity between the three types of cluster lifetimes. More specifically, if $L_{ab}(t,x,r_c)$ designates the lifetimes distributions of clusters between species *a* and *b* for a given concentration *x* of species *a*,and for cutoff we find that, while the curve shapes of the three species–species combinations differ somewhat markedly, the *x*-dependence is nearly the same as for the neat liquids. In other words, even in mixing conditions, these systems show the same universal cluster lifetimes observed in neat liquids.

## Results

**Figure 1:**
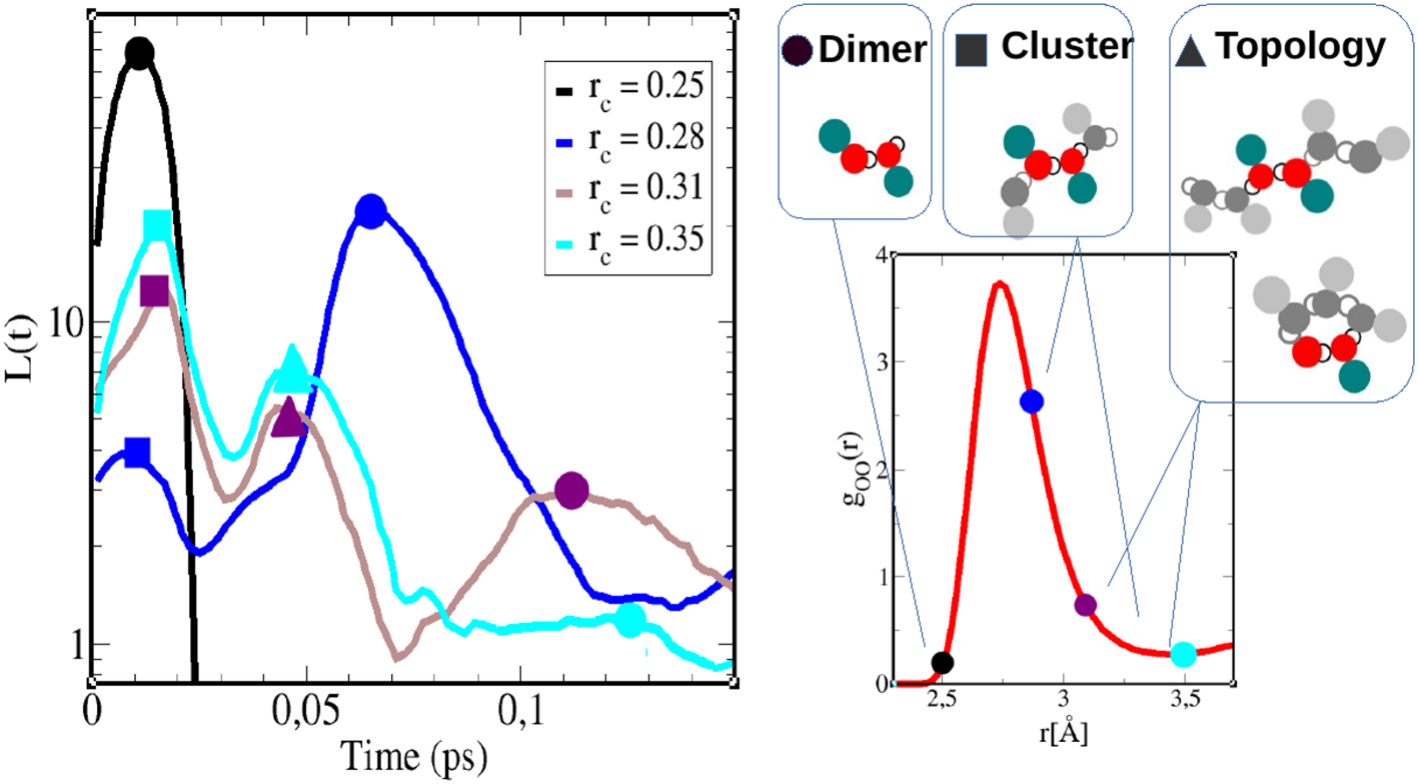
Illustration, for the case of pure methanol, of the correspondance between H-bond life time curves (left panel) for each bonding distances $r_c$ and their various peak/maxima (marked with symbols), with the dimer based clusters in relation with the oxygen–oxygen pair correlation function $g_{OO}(r)$ (right). The selected $r_c$ positions are marked on $g_{OO}(r)$ by the same color convention as in the left plot. Methanol molecule is modeled with red site for oxygen, open circle for hydrogen, and larger dark green site for the methyl groups. Only dimers are colored, while methanol molecules part of larger clusters are shown in grey shades. Refer to the text for additional explanations.

Figure 1 is an introductory reminder of the features reported in^12^, which should serve as a guidance to study the various mixtures studied herein.

In the left panel of this figure, typical H-bond life time *L*(*t*) curves for pure methanol (OPLS model) are plotted versus time, each for a given $r_c$ Hbonding distance cutoff. For shorter $r_c$ values, these curves have only one extremum (such as the black curve), or many extrema. The extrema which correspond to methanol dimers are highlighted with a filled circle, which those corresponding to larger clusters are highlighted with filled squares and triangles. On the right panel, the oxygen–oxygen pair distribution is shown in red curve, on which the selected $r_c$ distance from the left panel are shown as filled circles with same color conventions as the *L*(*t*) curves in the left panel. With these elements we are in position to interpret the various maxima of the *L*(*t*) curves, in terms of the cluster shapes highlighted on the top of the right panel. What is noticed is that, as $r_c$ is increased, the amplituded of the maximum marked with a filled circle diminishes, while its position moves to higher time values. When $r_c$ crosses the maximum of the $g_{OO}(r)$, 2 secondary maxima appear at lower times than the principal one, and we marked them with a filled square for the first one and a filled triangle for the second one. What we note is that, as $r_c$ is further increased, the position of these maxima do not change (up to statistical uncertainties), while their amplitude increases (in contrast to that marked with the open circle, which keeps on decreasing until it is no longer locatable). How to interpret these various maximum? In^12^, we have attributed the first maxima to lifetimes of the oxygen dimers, while the secondary and tertiary $r_c$ independent maxima where attributed respectively to larger clusters and to their shape/topology dependence. At this point, it is important to note that the *L*(*t*) curves, by definition, concern only dimers. For this reason, we have interpreted the principal maxima (filled circles) as corresponding to dimer lifetimes. What the *L*(*t*) curves show, is that, as we consider larger and larger $r_c$ distances, hence encompassing more and more neighbours, as their positions on the $g_{OO}(r)$ curve indicate, such dimers tend to live longer lifetimes, while the probability of their existence decreases. This seems intuitively reasonable. This base dimer is illustrated as colored set of circles in the upper row of the right panel, in the case of methanol molecules, where the open circle designates the hydrogen atom, the filled red circle is oxygen and the filled dark green circle is the CH3 methyl group. In this context, what could the secondary and tertiary maxima could correspond to? Our interpretation is that, as the cutoff $r_c$ is increased, *L*(*t*) accounts for larger clusters where the base dimer could be part of. Hence, there is an indirect signature of how the fact that these dimers are part of larger clusters, influence their stability, hence their lifetime. This is what the secondary maximum, highlighted by the filled square, indicates, and the fact that the amplitude increases with increasing cutoff $r_c$ seems consistent with the idea that larger clusters tend to stabilize better the base dimers contained in them. This is illustrated by a 4-member methanol cluster in the middle of the upper row of the right panel, where methanol molecules additional to the base one, are shown in grey shaded colors. Even though non-intuitive at first, this interpretion of the secondary peak appears reasonnable. But then, why a tertiary maximum would exist, which would appear at even larger lifetimes, albeit with lower amplitude to the secondary maximum? Our interpretation is that this tertiary peak corresponds to a topology of the cluster. This interpretation makes sense, since clusters come in various shapes. In the case of mono-ols such as methanol, many investigation show that chains, loops and lassos are found in various proportions. We can imagine that the lifetime and stability of dimers should differ, according to whether they are part of chain or loop cluster. Hence, this tertiary peak would correspond to the topology of the clusters, and this is illustrated on the right of top row of the right panel, with 2 typical chain and loop Hbonded clusters. For the OPLS methanol studied here, the peak positions of these secondary and tertiary lifetimes are found to be $\tau _2 \approx 0.02$ps and $\tau _3 \approx 0.05$ps, respectively. In Ref.^12^, we observed that these 2 values tend to remain the same across other types of Hbonded liquids based on the OH bonding. Theses values changed slightly in the case of nitrogen based Hbonding, as propylamine. These findings support the idea of an universality of clustering lifetimes in similar Hbonding liquids, irrerspective of their molecular nature. Finally, we note that there are no more distinct peak features beyond 0.1ps and *L*(*t*) decays algebraically^8^.

In the present work, we investigate what happens to these peaks in mixing conditions. It important to note that, although we found a universality of curve shapes and lifetimes, the curves reported in Ref.^12^ differ for different species. The results we show below indicate that these 3 features are preserved, even for the cross species bonding patterns. Since now the clusters contains cross species bonded molecules, this result is not obvious to predict.

### Water–methanol mixtures

**Figure 2:**
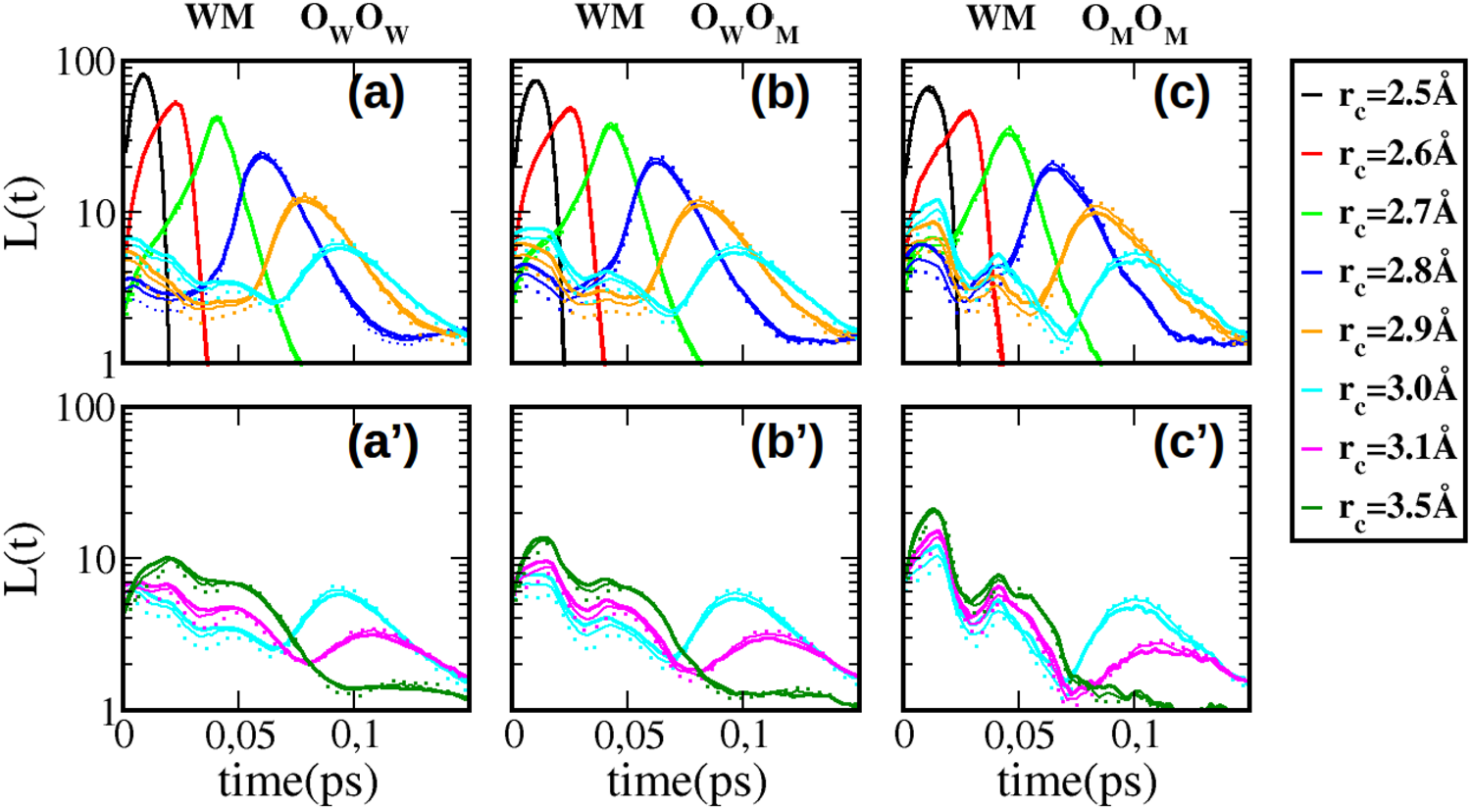
H-bond life time distributions *L*(*t*) for water–methanol (WM) mixtures, for (**a**,**a’**) water–water clusters, (**b**,**b’**) for water–methanol clusters, and (**c**,**c’**) for methanol–methanol clusters. The upper panels (**a**–**c**) show the primary peak features, while the lower panels (**a’**–**c’**) show the secondary and tertiary peak features. The thick full lines are for $x_{M}=0.2$, the thin lines for $x_{M}=0.5$, the dotted lines for $x_{M}=0.8$.

Figure 2 shows the lifetimes for the water–methanol (WM) mixtures, and for several $r_{c}$ distance cutoff values corresponding for different color codes (displayed on the far right panel). The 3 oxygen Hbonding possibilities are show each in 3 separate sets of upper and lower panels, namely those involving the oxygen of water ($\hbox {O}_{\mathrm{W}}$-$O_{\mathrm{W}}$) in panels (a) and (a’),the cross oxygen bonds ($\hbox {O}_{\mathrm{W}}$-$\hbox {O}_{\mathrm{M}}$) in panels (b) and (b’), and those between methanol oxygen ($\hbox {O}_{\mathrm{M}}$-$\hbox {O}_{\mathrm{M}}$ in panels (c) and (c’). For each $r_c$ specifically colored curve, 3 methanol concentrations are shown as thick line ($x=0.2$), thin line ($x=0.5$) and dotted line ($x=0.8$). The upper panels(a,b,c) show th primary life time curves, and the lower panels (a’,b’,c’) shown the secondary and tertiary lifetimes. This depiction allows to better visualise the specific features highligted in Fig. 1. The first striking feature is unmistakable simililarity between the 3 sets of panels, highly suggesting that that the *L*(*t*) features, described in the previous section, are the same in mixtures as in neat liquids. This similarity indicates that the lifetime *L*(*t*) is nearly insensitive to whether the owygen atoms belong to water or methanol. This is supported by the fact that the oxygen pair distribution functions are very similar across different methanol concentrations. In fact, this is generally the case across many systems having hydroxyl groups as base for hydrogen bonding. This is illustrated in Fig. 3B of the SI document. The second striking feature is that there is a very small concentration dependance of the lifetimes *L*(*t*), as can be seen by the proximity of different line types for a given color. This feature can equally be related to the near concentration independence of the first maximum and first minimum of the $g_{OO}(r)$ curves across several system having the OH hydroxyl group as Hbonding base (see Fig. 3B of the SI). These 2 features witness the fact that, within the classical force field models used in this work, the strong Coulomb interactions between charged atoms dominate the structural disposition of the molecules in a mild species segregation^18^. We now focus on the 3 features of the *L*(*t*) features briefly discussed in the previous section. As explained in^12^, but also in the introductory part to this section, the various curves shown in each set of upper/lower panels show 2 typical features. In the upper panels, a series of high peaks dominate the figures, whose maximum is seen to move to larger times with increasing $r_{c}$ values, as well as decreasing in amplitude. In addition, as shown in the lower panels, from $r_{c}$ values corresponding to crossing the maximum of the $g_{OO}(r)$ (see Fig. 1), each curve is seen to develop additional peaks at times smaller than the first peak and also having larger amplitudes. These peaks come in two, and we named them secondary and tertiary peaks. It is these peaks which correspond to the cluster modes. The secondary peak corresponds to clusters, while the tertiary peak would correspond to topology of these clusters (chain-like or globular-like, for example). For the present case of mixtures, it is seen that the first peaks show very little concentration dependence, with, however, an increase of dependence with larger $r_{c}$ values. The secondary and tertiary peaks show a somewhat stronger concentration dependence, in fact quite similar to that corresponding to the fact that they are for larger $r_{c}$ values. Let us try to rationalise the various trends observed in Fig. 2.

We first note that the variation of the concentration dependence of the primary peaks for water–water clusters (a) is opposite of that for methanol–methanol clusters (c). Namely, in Fig. 2a, these curves show an increase of magnitude with decreasing water concentration. It is the inverse in Fig. 2c: the magnitude increases with increasing methanol concentration. This behaviour is in fact exactly that of the first peak of the respective $g_{OO}(r)$. When water concentration decreases, water dimers gain strength. This can be witnessed by the fact that the first peak of $g_{O_{w}O_{w}}(r)$ increases when water concentration increases. Similarly, methanol first peak of $g_{O_{M}O_{M}}(r)$ is seen to decrease when methanol content decreases. This observation confirms that these primary peaks correspond indeed to water–dimers. Next, we observe that the secondary and tertiary peaks behave in opposite manner of their respective primary peaks. Indeed, in Fig. 2a’, the peaks are seen to decrease with water concentration, while in Fig. 2c’, they are seen to increase with decreasing methanol concentration. This indicates that water clusters become more loose with increasing methanol concentration, while methanol clusters become stronger when methanol is minority. This is similar to surfactant self-aggregating in water. Figure 2b,b’ shows that, from the primary peaks, water–methanol dimers increase in strength with increasing methanol content, but from secondary and tertiary peaks, that larger clusters decrease in strength with the increase of methanol content.

### Water–ethanol mixtures

**Figure 3:**
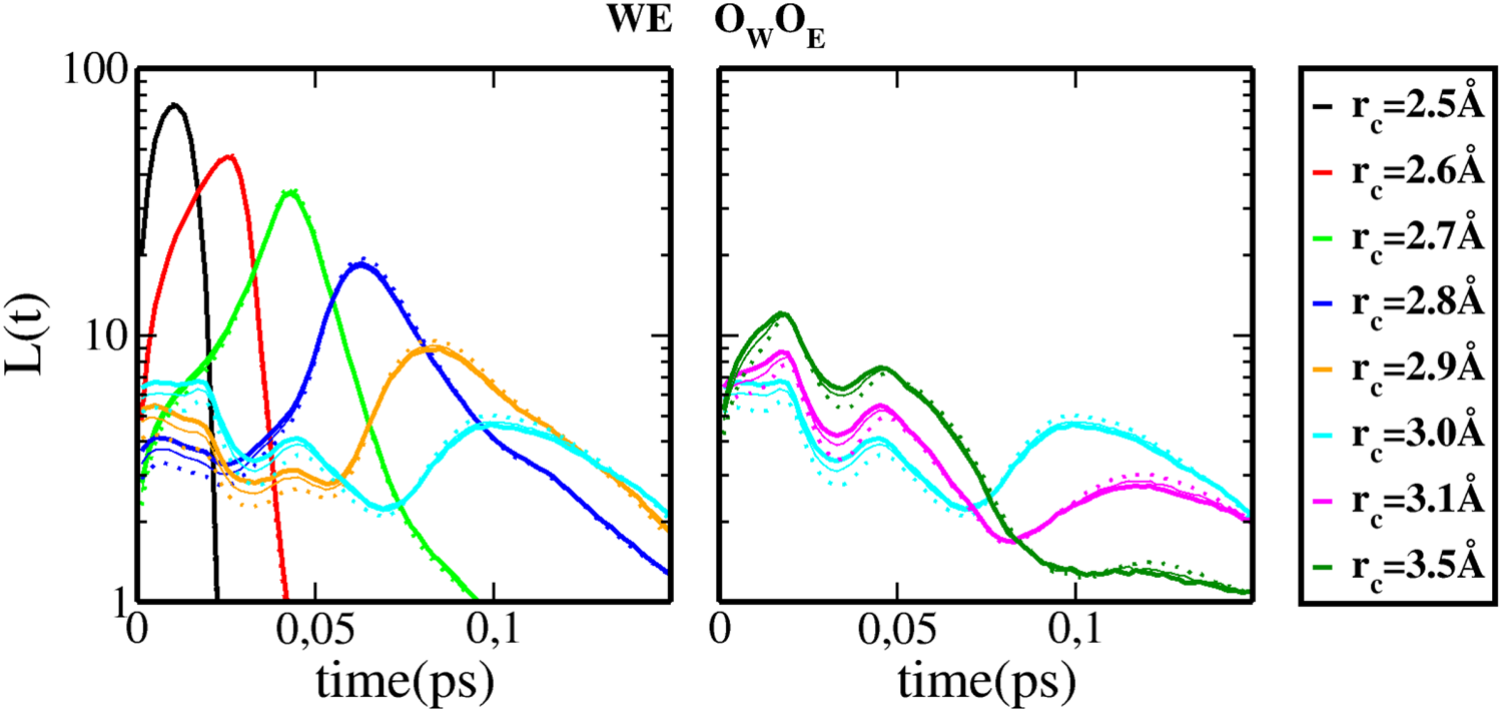
H-bond life time distributions *L*(*t*) of water–water clusters in water–ethanol (WE) mixtures. The full lines are for ethanol concentration $x_{E}=0.2$, the small dashes for $x_{E}=0.5$ and large dashes for $x_{E}=0.8$. Complementary informations in Fig. 1B of the SI document.

Next, we examine water–ethanol mixtures. We expect here to see how the extension of the alkyl tail influences the data observed in Fig. 2 for methanol. The equivalent of Fig. 2 is displayed as Fig. 1B of the SI document, and we focus here only the water–ethanol cross correlations. Figure 3 show trends very similar to Fig. 2, indicating that there are very little lifetime distribution differences, if we except the small *L*(*t*) curve shapes differences between methanol and ethanol. It tends to further confirm the idea of universal features introduced for pure liquids.

**Figure 4:**
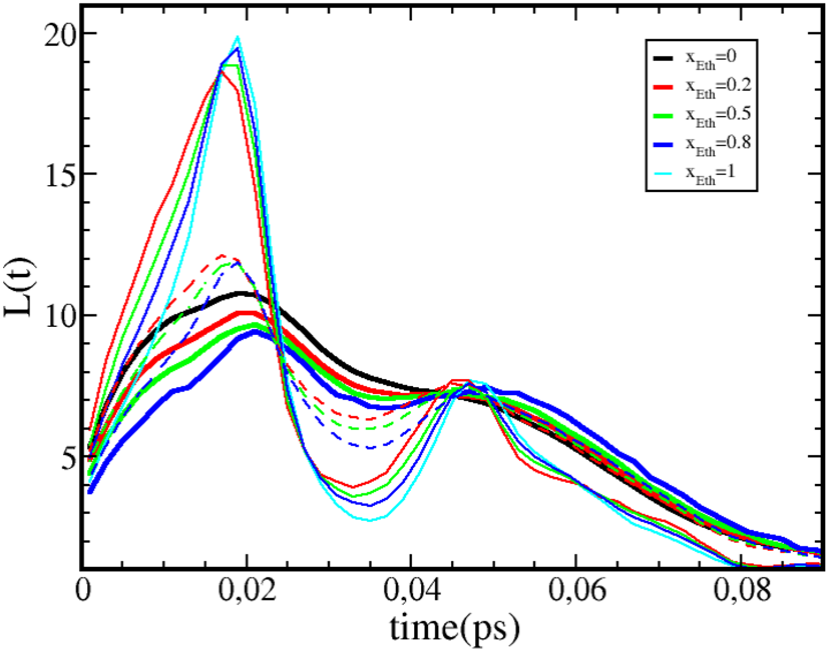
Close-up of the secondary and tertiary peaks of H-bond life time distributions *L*(*t*) of oxygen atoms for water–ethanol mixtures and for $r_c=3.5\mathring{A}$ and different ethanol concentrations. Water–water curves are in full lines, ethanol–ethanol lines in thin full lines, and water–ethanol cross contributions in dashed lines.

In order to illustrate both the differences and the universalities of the secondary and tertiary oxygen cluster lifetime features, we show in Fig. 4 a zoom on the corresponding curves for the water–ethanol mixtures selected for the cutoff value $r_c=3.5\mathring{A}$. On this figure, one observes clearly both the concentration dependence, which gathers all types of oxygen–oxygen correlation in 3 groups, namely $O_W O_W$, $O_W O_E$ and $O_E O_E$ pairs, as well as the very similar values for the lifetimes of the secondary and tertiary clusters types, that is $\tau _1 \approx 0.02$ps and $\tau _2 \approx 0.05$ps. The small differences are attributable to the fact that the $r_c$ value is fixed to $r_c=3.5\mathring{A}$, and may not quite correspond to that of the proper position of the various $g_{OO} (r)$ curves. This figure demonstrates the assertion of the universality of the features of *L*(*t*).

### Water–DMSO mixtures

**Figure 5:**
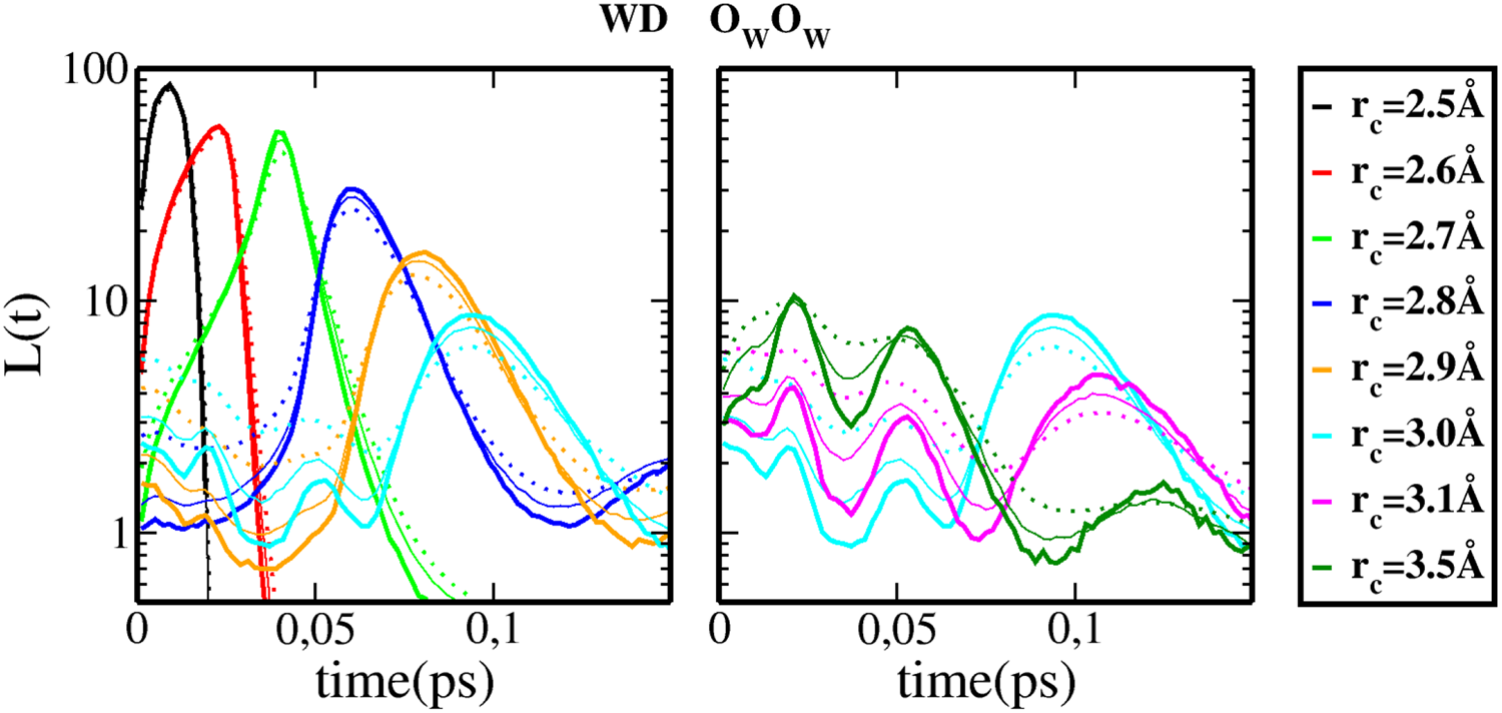
H-bond life time distributions *L*(*t*) for water–water oxygen contributions in water–DMSO mixtures. Line style versus solute concentrations are the same as in Figs. 2, 3. Complementary informations in Fig. 2B of the SI document.

Figure 5 shows selected lifetime distributions for water oxygen atoms (for the full information, refer to Fig. 2B in the SI document).

This mixture shows a feature not seen in previous aqueous mixtures. Indeed, while water oxygen–oxgen dimer lifetimes appear to follow patterns similar to that observed in previous aqueous mixtures, the cross oxygen dimers upper primary peaks have a two-bump feature. This two bump feature is absent from the secondary and tertiary peaks. Interpreted in a direct way, these two bumps could refer to the existence of dual water–DMSO dimer lifetimes, for a given $r_c$ value. From our previous study of aqueous–DMSO mixtures^24,25^, we speculate that this duality could arise from large sulfur atom, which creates a double first peak structure in the various oxygen atoms correlations. We note from Fig. 5, that there is a quite large concentration dependence of the secondary and tertiary peak amplitude, while the positions seem to obey the same universality as that observed in previous graphs, that is $\tau _1\approx 0.02$ps and $\tau _2\approx 0.05$ps.

### Alcohol–solute mixtures

**Figure 6:**
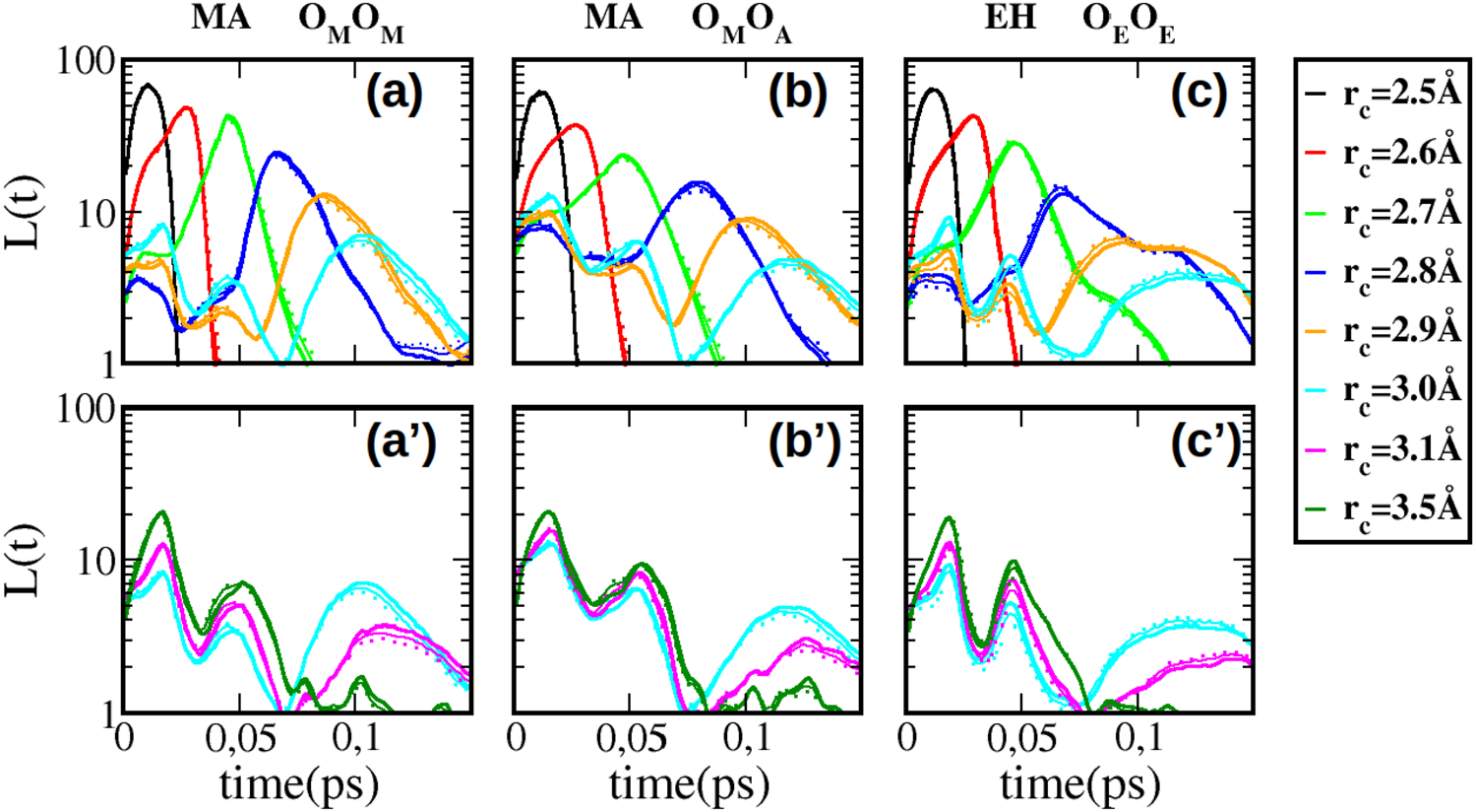
H-bond life time distributions *L*(*t*) for methanol–acetone (MA) and ethanol–hexane (EH) mixtures. Panel (**a**,**a’**) methanol–methanol clusters, (**b**,**b’**) for methanol–acetone clusters, and (**c**,**c’**) for ethanol–hexane clusters. The upper panels (**a**–**c**) show the primary peak features, while the lower panels (**a’**–**c’**) show the secondary and tertiary peak features. The thick full lines are for $x=0.2$, the thin lines for $x=0.5$, the dotted lines for $x=0.8$, where *x* is the alcohol content in the mixture.

We examine now the case of mixing alcohols with polar and non-polar solute. Figure 6a,b shows the lifetimes of the oxygen–oxygen Hbonding for the methanol–acetone mixture, while Fig. 6c shows the data for ethanol–hexane mixtures.

In the case of methanol–acetone, both Hbonds between methanol molecules, and those between methanol and acetone can be calculated, since acetone is only an acceptor of Hbonds. However, for the ethanol–hexane mixtures, ethanol is the only Hbonding species, as depicted in Fig. 6c,c’. Once again, all three panels of Fig. 6 show the same 3 peak characteristics observed for the 2 previous mixtures, and the single component liquids in^12^. However, it is interesting to compare methanol and ethanol self-bonding in presence of water and the solutes. Comparison of Figs. 2c and 6a show both the striking similarities in curve shapes for each $r_{c}$, but also the small inversion of peak behaviour with alcohol concentration. However, we note that in Fig. 6a, there is very little concentration dependence, even for the secondary and tertiary peaks. This observation is corroborated by our previous studies of methanol–acetone, where we observed that methanol tended to form same types of clusters as in pure liquid^26,27^. Similarly, we observe in Fig. 6b,b’ that methanol and acetone Hbonding distribution is equally nearly concentration independent. But the most important point here is that, even the Hbonding between an associating and non-associating species obeys the universality of time distribution. This finding confirms once more that these distributions are really about clusters, which are a permanent feature of the mixtures examined here. Finally, we note that ethanol Hbond lifetime distribution in hexane is nearly the same in water as in hexane, despite the very different properties of these two latter liquids. In addition, there appear to be no inversion of curves with concentration dependance.

### Long time kinetics

**Figure 7:**
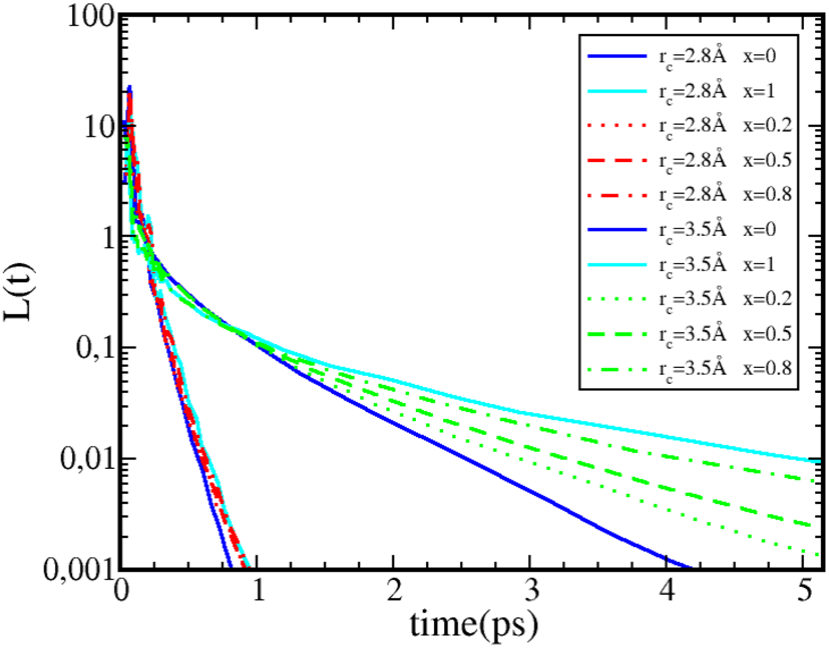
Cutoff and concentration dependence of the H-bond life time distributions *L*(*t*) for aqueous ethanol mixtures. Lines for cutoff $r_c=2.8\mathring{A}$ in red, and lines for cutoff $r_c=3.5\mathring{A}$ in green. Line for pure water in blue, and pure ethanol in cyan. Lines for ethanol concentration x=0.2 in dotted lines, for $x=0.5$ in dashed lines and for $x=0.8$ in dash-dotted lines.

Earlier works^8,9,28,29^ have emphasized that the long time behaviour of the lifetime *L*(*t*) should be more relevant to study of the hydrogen bond kinetics, the short time part being termed “transient regime”. Since in the present work we clearly show the important universality of this transient part, it is perhaps relevant to examine the long time part of *L*(*t*) in terms of both the cutoff $r_c$ and the concentration dependence for mixtures. In the previous work^12^ we have shown that the $r_c$ dependence was not relevant for $r_c$ values close to the first minimum of $g_{OO}(r)$ (see Fig. 1), since all first neighbour bonded pair were accounted for. Figure 7 shows *L*(*t*) function for the 3 oxygen–oxygen correlations in water–ethanol mixtures, for 2 different values of $r_c$, $r_c=2.8\mathring{A}$ and $r_c=3.5\mathring{A}$, and for ethanol concentrations $x=0$, $x=0.2$, $x=0.5$,$x=0.8$ and $x=1$.

As far as $r_c$ is concerned, we find the same convergence towards $r_c=3.5\mathring{A}$, the curves (dashed-dot) for the smaller distance $r_c=2.8\mathring{A}$ being clearly separated from the others in the lower left part of the graphs. In contrast, we find a strong concentration dependence of the lifetimes *L*(*t*), as the curves are seen to shift from pure water curves (in blue) to pure ethanol ones (in cyan) when ethanol concentration is varied. Interestingly, the proximity of the $O_W O_W$ curve for $x=0.2$ to the *OO* curve of pure ethanol reveals that the long time kinetics of water oxygen are slaved to that of the neighbouring ethanol oxygen, suggesting the existence of cross oxygen clusters. Similar remarks can be made for low ethanol content in water, and also for the cross osygen correlations. The concentration dependence shown in Fig. 7 is not surprizing, as one expects the hydrogen bond kinetics to depend on the composition of the mixtures. A similar dependence was equally observed in the amplitudes of the cluster peaks reported in the previous sections. However, these dependences do highlight the invariance of the secondary and primary peaks positions of *L*(*t*) as a highly non-trivial feature of the hydrogen bond clustering.

## Discussion

The present study underlines the fact that the Hbond based clustering has very similar basis across different Hbonding species and atoms. This is particularly important in mixtures, despite the fact that Hbonding differences in interactions imposes a micro-segregation of the species^18,19,30,31^. Both in single component or mixtures, the elementary unit is the pair of Hbonding molecules. The present study confirms the universal character of this Hbonding, since it is essentially based on Coulomb pairing at the level of the description in computer simulations. The fact that simulations based on classical force fields are able to reproduce satisfactorily thermo-physical, dynamical and scattering properties of many systems^32–37^, is a strong support to the validity of the findings of the present study. The secondary and tertiary peak features of the lifetime are based on the pair representation. The present study shows that these larger clusters obey very similar patterns even in mixing conditions. This is important for understanding and interpreting molecular binding properties in realistic systems, particularly at the short time scales where chemical reactions occur usually, which is the case with mixtures. This is why the present study may be important in confirming what was already found in the case of pure liquids. Finally, the existence of an universal secondary and tertiary lifetime peaks have an appealing conceptual link to the concepts of local energy and entropy. Indeed, clustering is essentially an energy based process, akin to a labile equivalent of a covalent bonding. Since for a given cluster size many cluster shapes can exist, there is an entropical dimension to the clustering process. One may wonder if these 2 concepts of local energy and entropy are not abstractions. Our findings indicate that, since there is a specific distinct lifetime peak associated with each of these local manifestations, the idea of local energy and local entropy have a realistic physical interpretation, which deserve subsequent investigations.

## Methods

The main physical quantity introduced and examined in our previous work^12^ was lifetime distribution of hydrogen bonds in different types of pure associative liquids. In the present work we extend our research to the mixtures listed in the Introduction. Again, purely geometric criteria has been used in order to detect a hydrogen bond in the system, meaning that a random pair of molecules *i* and *j* is considered to be hydrogen bonded if the distance $r_{ij}$ between the corresponding donor and acceptor atoms ($A_i$ and $B_j$) satisfies $r_{ij} \le r_C$ and if the angle $\theta _{ij}=\angle {B_jA_iH}$ obeys $\theta _{ij} \le \theta _C$. The critical values $r_C$ and $\theta _C$ are equal to 3.5 Å and $30^{\circ }$ respectively. The probability distribution of hydrogen bonds *L(t)* is properly defined in our previous work^12^ and the same definition is used here. *L(t)* has been calculated using module *gmx hbond* (with *-life* option) which is available in GROMACS package^38,39^.

Likewise, all of the simulations were executed in the GROMACS program package^38,39^. The initial configurations were generated using the Packmol software^40^, with the majority of studied systems containing 16000 molecules. The systems were first energy minimized and then equilibrated for a total of 1 ns. Production runs were performed after the equilibration, lasting 1 ns. Furthermore, we used the leap-frog integrator^41^ with the timestep of 2 fs. The short-range interactions were calculated within the 1.5 nm cut-off radius. The particle mesh Ewald (PME) method^42^ was used for handling the long range electrostatic calculations, with the FFT grid spacing of 0.12 nm and the interpolation order of 4. The LINCS algorithm^43^ was used to constraint the length of intramolecular bonds. All simulations were performed in the isobaric-isothermal (NPT) ensemble. The temperature was held fixed at T=300 K by using the Nose–Hoover thermostat^44,45^ with the time constant of 0.1 ps and the pressure was kept at p=1 bar with the Parrinello–Rahman barostat^46,47^ with the time constant of 1 ps.

We used SPC/E^48^ and TIP4P_2005^49^ forcefields to simulate water and OPLS-UA^50^ and TraPPE-UA^51^ forcefields to simulate alcohols. Propylamine was simulated using OPLS-AA forcefield^52^.

## Supplementary Information

Supplementary Information.
