# Supplementary material for: Lifetime distribution of clusters in binary mixtures involving hydrogen bonding liquids
Source: Sci Rep. 2022 Jun 1;12:9120. doi: 10.1038/s41598-022-12779-0 (PMC9160284; doi:10.1038/s41598-022-12779-0)
Supplement: Supplementary file 1 — Supplementary Information. [file 41598_2022_12779_MOESM1_ESM.pdf]

SUPPORTING INFORMATION

Universal features in Lifetime Distribution of Clusters in Hydrogen Bonding Liquids

Ivo Jukić[‡], Martina Požar[‡], Bernarda Lovrinčević[‡] and Aurélien Perera[†]

[†]Laboratoire de Physique Théorique de la Matière Condensée (UMR CNRS 7600), Sorbonne Université, 4 Place Jussieu, F75252, Paris cedex 05, France.
[‡] University of Split, Faculty of Science, Ruđera Boškovića 33, 21000, Split Croatia.

A – Definition of the lifetime function $L(t)$

We consider 2 hydrogen bonding molecules, one having an acceptor site X1 and the other a donor site X2 (typically oxygen or nitrogen). These 2 molecules are said to be hydrogen bonded when the pair of sites (X_1,H_1) and (X_2,H_2) is positioned in such a way that the distance between X_1 and X_2 is less that a cutoff-distance r_c , and the angle between $\overrightarrow{X_1 H_1}$ and $\overrightarrow{X_1 X_2}$ is less than an angular cutoff θ_c . For a given pair of Hbonding molecules i and j , if they first enter in bonding at time τ_1 and leave the bonding at time τ_2 , their Hbonding life time is $\tau_{ij}=\tau_2-\tau_1$. We can use a Dirac delta function to define a microscopic lifetime random variable as:

$$h_{ij}(t)=\delta(t-\tau_{ij}) \tag{1A}$$

This new definition replaces that used in our previous work Ref.[1]. Once such a random variable is defined, one can perform statistics on it, for example within a computer simulation of hydrogen bonding liquids and mixtures. This variable will be dependent on the choices of the cutoff r_c and θ_c .

The lifetime function $L(t)$ can be defined from the histogram

$$L(t)=\frac{1}{L_0}\sum_{ij\in\mathscr{L}} h_{ij}(t) \tag{2A}$$

where the sum runs over the ensemble $\mathscr{L}$ of all the bonded pairs (i,j) at a given time t , and the normalization L_0 is defined such as to guaranty that, for a fixed set of (r_c , θ_c) we have

$$\frac{1}{T_0}\int_0^{T_0} dt\, L(t)=1 \tag{3A}$$

where T_0 is the simulation run time length, and this condition implies the definition

$$L_0=\frac{1}{T_0}\int_0^{T_0}\sum_{ij\in\mathscr{L}} dt\,\delta(t-\tau_{ij}) \tag{4A}$$

Since the integral is just the number of Hbonding events occurring in the simulation interval $[0,T_0]$, L_0 is akin to the frequency of Hbonding events.

In the calculations, the Hbonding angle is set at 30 degree, as in Ref.[1], and it is the bonding distance r_c which is varied.

B – Aqueous mixtures

Below, the entire data for all 3 oxygen bonding possibilities of the ethanol-water mixtures are displayed in Fig.1B. The cyan curve corresponding to $r_c=3\,\mathring{A}$ is reproduced in both the upper and lower panels, in order to ensure visual continuity of the display across the separation of the

primary (upper panels) and secondary/tertiary (lower panels) peaks discussed in the main text.

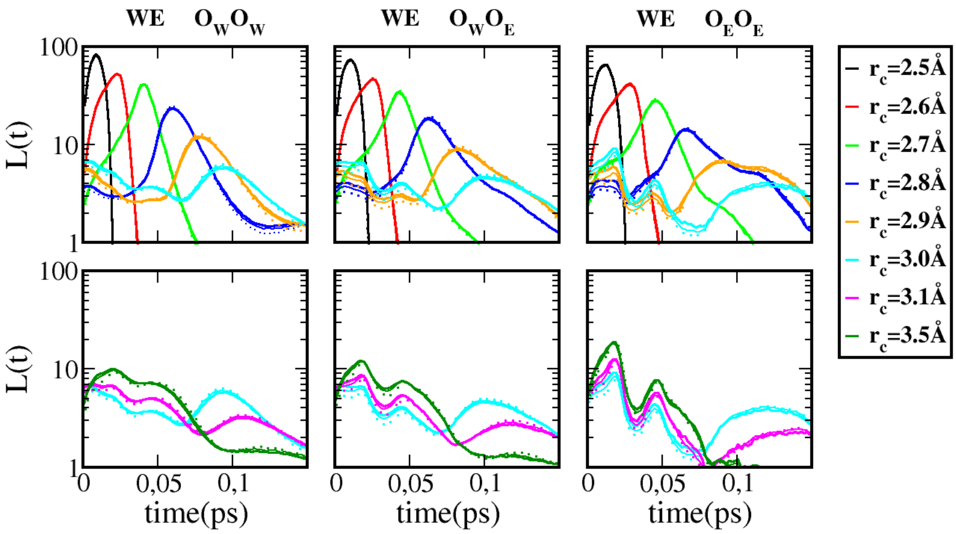

Fig.1B. Hydrogen lifetimes for the aqueous ethanol mixtures (refer to Fig.3 of main text), as a function of the cutoff distances (displayed on the far right panel with appropriate line color codes). The same conventions as Fig.3 of the main text are used. Each set of vertical panels correspond to one type of oxygen pairs: water-water (O_WO_W), water-ethanol(O_WO_E) and ethanol-ethanol(O_EO_E). The upper panels are for primary lifetimes, and the lower panels for secondary lifetimes.

The entire information for $L(t)$ of the water-DMSO mixtures is shown below

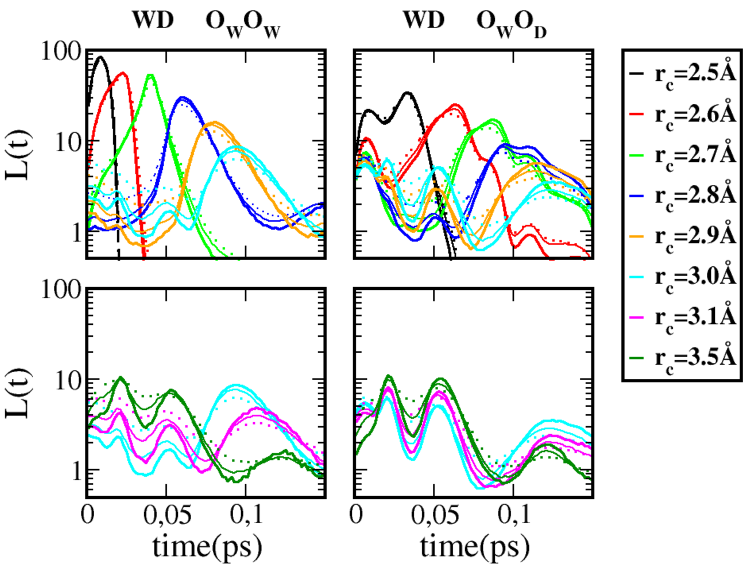

Fig.2B. Hydrogen lifetimes for the aqueous DMSO mixtures (refer to Fig.5 of main text), as a function of the cutoff distances (displayed on the far right panel with appropriate line color codes). The same conventions as Fig.1B above of the SI are used.

Fig.3B illustrates the fact that, for all Hbonding liquids based on the OH hydroxyl group, the first maximum and first minimum of the oxygen-oxygen distribution functions have nearly the same r-positions across different systems, both neat and mixtures. This is certainly in relation with the fact that both secondary and tertiary lifetimes $\tau_1 \approx 20$ fs and $\tau_2 \approx 50$ fs are similar across different such systems.

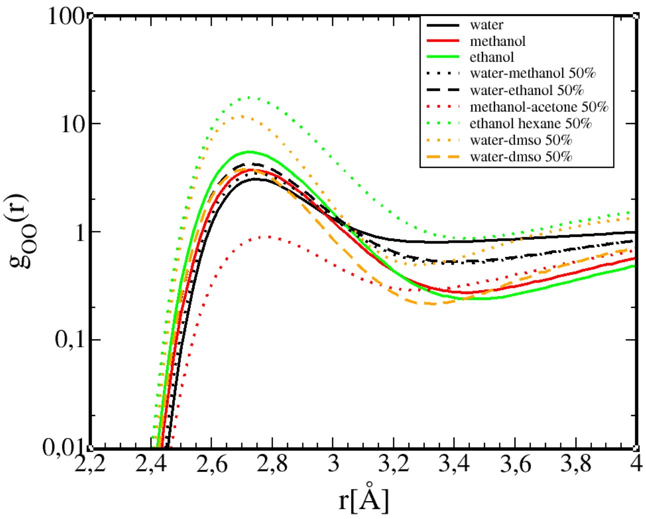

Fig.3B. Oxygen-oxygen radial distribution functions (logscale) for various systems (neat and mixtures). This figure serves to demonstrate that both the main peak and first minimum are quite similar across many systems having hydroxy group in common.

REFERENCES

[1] Jukić, I., Požar, M., Lovrinčević, B. & Perera, A. Universal features in the lifetime distribution of clusters in hydrogen-bonding liquids. Phys. Chem. Chem. Phys. 23, 19537–19546, DOI: 10.1039/D1CP02027G (2021).
